# Enhanced and long-lasting SARS-CoV-2 immune memory in individuals with common cold coronavirus cross-reactive T cell immunity

**DOI:** 10.3389/fimmu.2025.1501704

**Published:** 2025-03-21

**Authors:** David M. Florian, Michael Bauer, Amelie Popovitsch, Ingrid Fae, David N. Springer, Marianne Graninger, Marianna Traugott, Lukas Weseslindtner, Stephan W. Aberle, Gottfried Fischer, Michael Kundi, Karin Stiasny, Alexander Zoufaly, Samuel J. Landry, Judith H. Aberle

**Affiliations:** ^1^ Center for Virology, Medical University of Vienna, Vienna, Austria; ^2^ Department of Transfusion Medicine and Cell Therapy, Medical University of Vienna, Vienna, Austria; ^3^ Department of Medicine IV, Klinik Favoriten, Vienna, Austria; ^4^ Center for Public Health, Department for Environmental Health, Medical University of Vienna, Vienna, Austria; ^5^ Faculty of Medicine, Sigmund Freud University, Vienna, Austria; ^6^ Department of Biochemistry and Molecular Biology, Tulane University School of Medicine, New Orleans, LA, United States

**Keywords:** SARS-CoV-2, mRNA vaccination, CD4 T cell response, cell-mediated immunity, long term immunity

## Abstract

With the continuous emergence of novel SARS-CoV-2 variants, long-lasting and broadly reactive cellular and humoral immunity is critical for durable protection from COVID-19. We investigated SARS-CoV-2-specific T cell immunity in relation to antibodies, infection outcome and disease severity and assessed its durability in a longitudinal cohort over a three-year time course. We identified pre-existing T cells reactive to the seasonal coronavirus (CoV) OC43 that cross-react with the conserved SARS-CoV-2 spike S_813-829_ peptide. These cross-reactive T cells increased in frequency following SARS-CoV-2 infection or vaccination and correlated with enhanced spike-specific T cell responses and significantly reduced viral loads. Furthermore, our data revealed that CoV-cross-reactive T cells were maintained as part of the long-lasting memory response, contributing to increased T cell frequencies against omicron variants. These findings suggest a functional role of CoV-cross-reactive T cells that extends beyond the initial SARS-CoV-2 exposure, contributing to enhanced immunity against highly mutated SARS-CoV-2 variants.

## 1 Introduction

The COVID-19 pandemic has highlighted the importance of characterizing immune responses against SARS-CoV-2 to understand its role in protection and vaccine efficacy. SARS-CoV-2 vaccination and infection elicit broad activation of B cells and T cells, which are essential for recovery and protection against COVID-19 (1–5). However, SARS-CoV-2-specific antibody responses wane over the first months (6, 7) and exhibit diminished neutralizing efficacy against antigenically drifted omicron variants, leading to reduced protection against infection (8–14). In contrast, memory T cells appear to be more durable (6, 15), display largely preserved reactivity against epitopes in antigenically distant strains and thus likely play important roles in protecting against severe disease with new SARS-CoV-2 variants, in particular in the context of weak neutralizing antibody responses (13, 16–19).

Since the early stages of the pandemic, it has been reported that cross-reactive T cells to SARS-CoV-2 epitopes were detected in SARS-CoV-2-naïve subjects (20–23) and were widely present in SARS-CoV-2-exposed, but PCR-negative individuals, suggesting that pre-existing T cell immunity is associated with protection from detectable infection (1, 24, 25). Part of this reactivity was ascribed to memory T cells recognizing epitopes in conserved regions between seasonal coronaviruses (CoVs) and SARS-CoV-2, such as those in the spike S2 domain (21, 24, 25). These sites harbor broadly recognized epitopes that induce immunodominant T cell responses in COVID-19 patients (3). In addition, it was shown that pre-existing T cells are associated with protection against infection and severe disease, and are able to confer faster and stronger immune responses to COVID-19 mRNA vaccines (1, 3, 21, 26–31). Given the ongoing emergence of novel SARS-CoV-2 variants with unprecedented evasion from neutralizing antibodies, pre-existing T cell immunity from prior infection or vaccination becomes increasingly important; however, we still lack a detailed understanding of the relevance of broadly cross-reactive T cells in the context of repeated SARS-CoV-2 exposures. In this study, we assessed the influence of CoV-cross-reactive T cell immunity on the strength and durability of SARS-CoV-2 T cell responses, and its implications for infection outcome and disease severity. Since T cell specificity and cross-reactivity are highly dependent on the individual human leucocyte antigen (HLA) background (32, 33), the study was performed in HLA-matched cohorts. Our results indicate that spike cross-reactive T cells enhance SARS-CoV-2-specific T cell responses and contribute to long-lasting memory responses, capable of recognizing CoV OC43 and highly mutated omicron variants, including BA.5 and XBB.1.5.

## 2 Materials and methods

### 2.1 Study cohort

**Figure 1:**
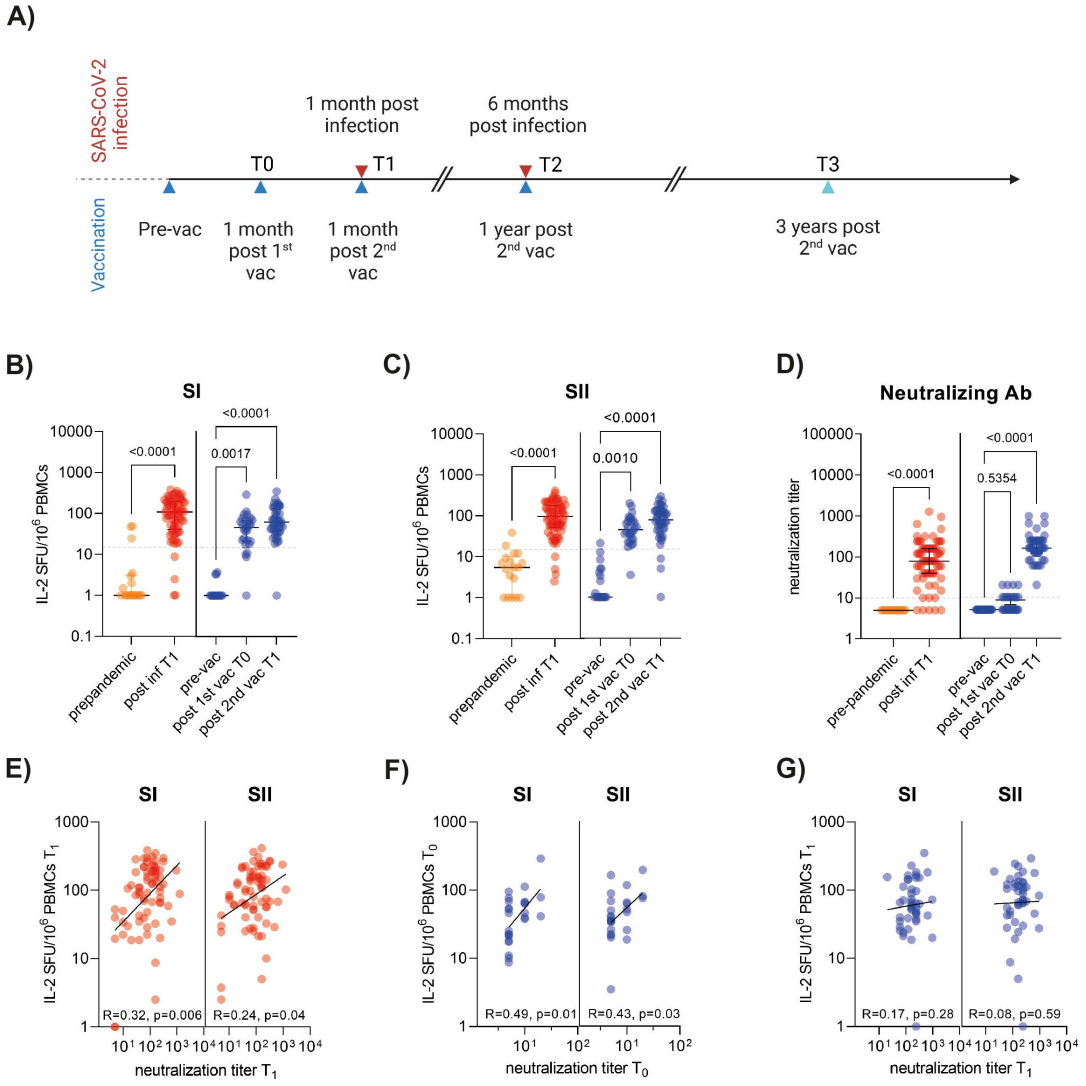
SARS-CoV-2 T cell responses to spike SI and SII in patients and SARS-CoV-2 naïve individuals after BNT162b2 vaccination. **(A)** Timeline of the study indicating blood sample collection points following vaccination and infection. Created with BioRender.com. **(B, C)** *Ex vivo* interleukin (IL)-2 ELISpot results after stimulation of PBMCs with SI and SII peptide pools covering the ancestral SARS-CoV-2 spike in pre-pandemic unexposed donors (n=18), COVID-19 patients (n=72) and BNT162b2 vaccinated individuals (n=44). **(D)** Neutralizing antibody titers, as determined by life-virus neutralization assays. Titers below the limit of detection (<10) were set to 5. The horizontal lines indicate median, with error bars illustrating the interquartile range (IQR). **(E-G)** Correlation between IL-2^+^ T cell responses to SARS-CoV-2 SI and SII peptide pools and neutralizing antibody titers after infection (T1) **(E)** or first (T0) **(F)** and second (T1) vaccination **(G)**. Each symbol represents one donor. The p-values represent the results of unpaired t-tests **(B-D)** and Spearman correlation **(E-G)**. Dotted lines indicate 15 SFU/10^6^ PBMCs. SFU, spot forming units.

The study cohort consisted of 72 COVID-19 patients (27 female, 45 male; age range, 21-80 years), including 52 subjects hospitalized with severe COVID-19 and 20 individuals with mild COVID-19 who tested positive for SARS-CoV-2 RNA by reverse transcriptase quantitative polymerase chain reaction from nasopharyngeal swabs. Blood samples were obtained between March 2020 and February 2021 at 1 month and 6 months post infection (**Supplementary Table 1**, **Figure 1A**). Blood samples from 44 individuals were obtained before and after the first and second COVID-19 mRNA vaccine dose. Blood samples from 18 healthy controls were collected between 2013 and 2019 through routine screening of blood donations performed by the Austrian Red Cross for prior unrelated studies. The donors were considered unexposed controls, given that the early phase of the first COVID-19 wave in Austria occurred in the spring of 2020 (34). The analysis of cross-reactive T cells was conducted in a subcohort of participants (“HLA matched cohort”, **Supplementary Table 1**) who carried specific HLA-DP or HLA-DR alleles that were previously shown to bind CoV-cross-reactive SARS-CoV-2 S_813-829_ and S_1002-1018_ peptides (35). Follow-up samples were obtained 1 and 3 years after the 2^nd^ vaccine dose (**Supplementary Table 1**, **Figure 1A**). Details of the follow-up cohort (vaccine types, SARS-CoV-2 omicron variants) are provided in **Supplementary Table 7**.

### 2.2 Detection of SARS-CoV-2 RNA

SARS-CoV-2 RNA was extracted from respiratory specimens using NucliSENS easyMAG extractor (BioMérieux, Marcy l’Etoile, France). SARS-CoV-2 real-time TaqMan PCR was performed as described previously (36).

### 2.3 Omicron variant identification

Nasopharyngeal swabs from routine diagnostics were PCR-tested for SARS-CoV-2. Variants were identified using Vir-SNiP SARS-CoV-2 assays (TIB MOLBIOL, Berlin, Germany) to distinguish between BA.1, BA.2, BA.5 and XBB.1.5. In some cases, variants were additionally confirmed by next-generation sequencing using amplification with ARTIC network tiled amplicon primers followed by Nextera XT library preparation and sequencing on an Illumina MiSeq device.

### 2.4 Sample collection and processing

Plasma was separated from whole blood samples by centrifugation for 10 min at 3000g and stored at -20°C until further use. Peripheral blood mononuclear cells (PBMCs) were isolated by density gradient centrifugation and stored in liquid nitrogen until further use. HLA typing of PBMCs was carried out by next generation sequencing, as described previously (37, 38).

### 2.5 *In-silico* MHC-II: peptide binding predictions

Predictions were performed using NetMHCIIpan4.1EL from the IEDB database (39), and percentile rank was selected as an output. Predictions were done for experimentally validated S_813-829_ (SKRSFIEDLLFNKVTLA) and S_1002-1018_ (QSLQTYVTQQLIRAAEI) peptides with the HLA II alleles from all individuals (**Supplementary Table 2**). Peptide:MHCII combinations with a percentile rank score >2% that were present in ≥10% of the study cohort were selected for further analysis.

### 2.6 Peptides

For T cell stimulation, Peptides S_813-829_ (SKRSFIEDLLFNKVTLA) and S_1002-1018_ (QSLQTYVTQQLIRAAEI) as well as pools containing non-conserved SARS-CoV-2-specific peptides were obtained through BEI Resources, NIAID, NIH: Peptide Array, SARS-Related Coronavirus 2 Spike (S) Glycoprotein, NR-52402. PepMix™ SARS-CoV-2 peptide pools covering the entire sequences of the SARS-CoV-2 spike proteins of the ancestral strain as well as omicron BA.4/BA.5, and XBB.1.5 variants (product codes: PM-WCPV-S-1, PM-SARS2-SMUT15-1 and PM-SARS2-RBDMUT10-1) were purchased from JPT (Berlin, Germany). The peptide libraries comprise 15-mer peptides overlapping by 11 amino acids (including a few 17-mers). Spike responses were tested in two separate pools, SI and SII, to distinguish responses to the N-terminal spike (covered by the SI peptide pool) from the more conserved C-terminal part (covered by the SII peptide pool), which includes peptides S_813-829_ and S_1002-1018_.

### 2.7 IL-2 ELISpot assay

ELISpot assays were performed as previously described (38, 40, 41). In brief, MultiScreen HTS IP Sterile Plates (Millipore) were coated with 1µg anti-human IL-2 antibody (3445-3-250, Mabtech) overnight and blocked with 5% BSA. PBMCs (200,000 cells/well) were incubated at 37°C and 5% CO2 for 20 hours with SARS-CoV-2 peptides (2 µg/ml; duplicates), AIM-V medium (negative control; 3-5 wells) or leucoagglutinin (PHA-L; L4144, Sigma; 0.5 µg/ml; positive control). Plates were washed and spots developed with biotinylated anti-IL-2 antibody (3445-6-250; Mabtech), streptavidin-coupled alkaline phosphatase (ALP; 3310-10, Mabtech; 1:1000) and 5-bromo-4-chloro-3-indolyl phosphate/nitro blue tetrazolium (BCIP/NBT; B5655, Sigma). Spots were counted using the Bio-Sys Bioreader 5000 Pro-S/BR177 and Bioreader software generation 10. The cut-off for positivity was >15 SFU/10^6^ PBMCs after background subtraction (mean spot count from 3-5 unstimulated wells).

### 2.8 Flow cytometry assays following 10-day *in vitro* stimulation

Flow cytometry and propagation of PBMCs was conducted as described previously (42). Briefly, PBMCs were stimulated with S_813-829_ (SKRSFIEDLLFNKVTLA) or the homologous OC43 S_911-927_ (RSAIEDLLFDKVKLSDV) peptide and cultured for 10 days adding 100 IU IL-2 on day 5. *In vitro* expanded cells were analyzed by intracellular cytokine and cell surface marker staining. PBMCs were incubated with 2 μg/ml of S_813-829_ (SKRSFIEDLLFNKVTLA) or the homologous OC43 peptide S_911-927_ (RSAIEDLLFDKVKLSDV) and 1 μg/ml anti-CD28/49d antibodies (L293 and L25, Becton Dickinson) or without antigen (negative control) for 6h. After 2h, 0.01 μg/ml brefeldin A (Sigma) was added. Tubes were stored in the freezer overnight. Cells were stained with APC/H7 anti-human CD3 (SK7, Becton Dickinson), Pacific Blue anti-human CD4 (RPA-T4, Becton Dickinson), and PE anti-human CD8 (HIT8a, Becton Dickinson). After surface staining, cells were fixed and permeabilized using a Caltag Laboratories Fix&Perm^®^ cell permeabilization kit (Invitrogen) as recommended by the manufacturer. Following permeabilization, cells were stained with APC anti-human IL-2 (5.34411e+006, Becton Dickinson), FITC-anti-human IFN-γ (25723.11, Becton Dickinson) and PE-CY7-anti-human TNF (MAB11, Becton Dickinson). Cell viability was assessed using ViViD Aqua Dead (Invitrogen). Boolean gating function of FlowJo was used to assess each cytokine combination. Responses obtained by Boolean analysis were considered positive if they were ≥ twofold above samples with no antigen stimulation. Frequencies below the cut-off for positivity were set to 10^-3^. Analysis was carried out using FACS Canto II (Becton Dickinson) and data processed using the FlowJo software v. 10.7.2 (Becton Dickinson).

### 2.9 Neutralization tests

Neutralization tests were performed as described previously (10). In short, duplicates of 2-fold diluted heat-inactivated serum were incubated with 50–100 TCID_50_ SARS-CoV-2 for 1h at 37°C and then added to a monolayer of Vero E6 cells. After incubation for 3-5 days, the cytopathic effect of non-neutralized infectious virus was assessed, and neutralization titers were assessed as the reciprocal of the serum dilution for protection against induction of cytopathic effect. The threshold for positivity was set at a neutralization titer of ≥10.

### 2.10 Statistical analysis

Statistical analysis was performed using Stata 17 (StataSoft, College Station, TX, USA) and GraphPad Prism 9 (GraphPad Software, Boston Massachusetts USA). For medians and post-vaccination neutralization titers, values <10 were set to 5. Unpaired t-test and one way ANOVA was performed for group comparison, the Spearman rank test was used for correlations, and the Wilcoxon matched-pairs test and Dunn´s multiple comparisons test for paired samples. Univariate analysis examined demographic and immunological parameters as predictors of viral load and disease severity. A multivariate linear or logistic regression model was used to examine the adjusted effect of T cells (independent variable), viral load and hospitalization (dependent variable), respectively. Generalized Structural Equation Models were applied with hospitalization as a Bernoulli variable, and age, log transformed viral RNA load and T cell counts as normal variates. For all analyses, a p-value <0.05 was considered significant.

### 2.11 Ethical approval

Studies were conducted in accordance with the Declaration of Helsinki in terms of informed consent and approval by the institutional review board of the Medical University of Vienna, Austria. All patients provided written informed consent. The use of biobanked plasma and cell samples from healthy pre-pandemic blood donors, patients and vaccinees, as well as virological data received by the Center for Virology has been approved by the Ethics committee of the Medical University of Vienna, Austria (EK 2156/2019, EK 2283/2019 and 1291/2021). Anonymized leftover samples were stored in the biobank of the Center for Virology, following established protocols (EK 1513/2016) in accordance with national legislation that required no additional written informed consent.

## 3 Results

### 3.1 SARS-CoV-2 specific T cell responses correlate with neutralizing antibody titers

We first determined the overall extent of SARS-CoV-2 T cell responses in *ex vivo* IL-2 ELISpot assays using peptide libraries composed of overlapping 15-mer peptides covering the full-length of spike protein in two pools SI and SII. The rationale for using an IL-2 ELISpot assay was based on previous studies, which demonstrated that IL-2 is a highly sensitive read-out to measure SARS-CoV-2 T cells associated with protection against infection (1), and on initial experiments indicating that IL-2 and IFN-γ assays detected similar quantities of SARS-CoV-2 T cell responses. We analyzed peripheral blood mononuclear cells (PBMCs) from 44 SARS-CoV-2-naïve individuals who received BNT162b2-mRNA vaccination, and 72 COVID-19 patients as well as 18 pre-pandemic controls (for characteristics of study participants, see **Supplementary Table 1**). The analysis revealed a significant increase in the levels of IL-2-secreting T cells (herein referred to as IL-2^+^ T cells) against SI and SII in both SARS-CoV-2-infected and vaccinated cohorts (**Figures 1B, C**). Virus-neutralizing antibodies, determined in live-virus neutralization assays, were detected in sera from 93% COVID-19 patients, and neutralizing titers correlated with IL-2^+^ T cell reactivity against spike SI and SII (**Figures 1D, E**). In vaccinated individuals, neutralizing antibodies were detected in 43% after the 1^st^ and 100% after the 2^nd^ vaccination (**Figure 1D**). IL-2^+^ T cell levels present after the 1^st^ vaccine dose positively correlated with neutralizing titers after the first dose (**Figure 1F**), while no correlation was found between neutralizing titers and IL-2^+^ T cell frequencies present after the second vaccine dose (**Figure 1G**). These findings are consistent with previous studies (41, 43), suggesting a role of antigen-specific T cells in facilitating the development of SARS-CoV-2-specific antibody responses.

### 3.2 SARS-CoV-2 cross-reactivity in healthy pre-pandemic donors

**Figure 2:**
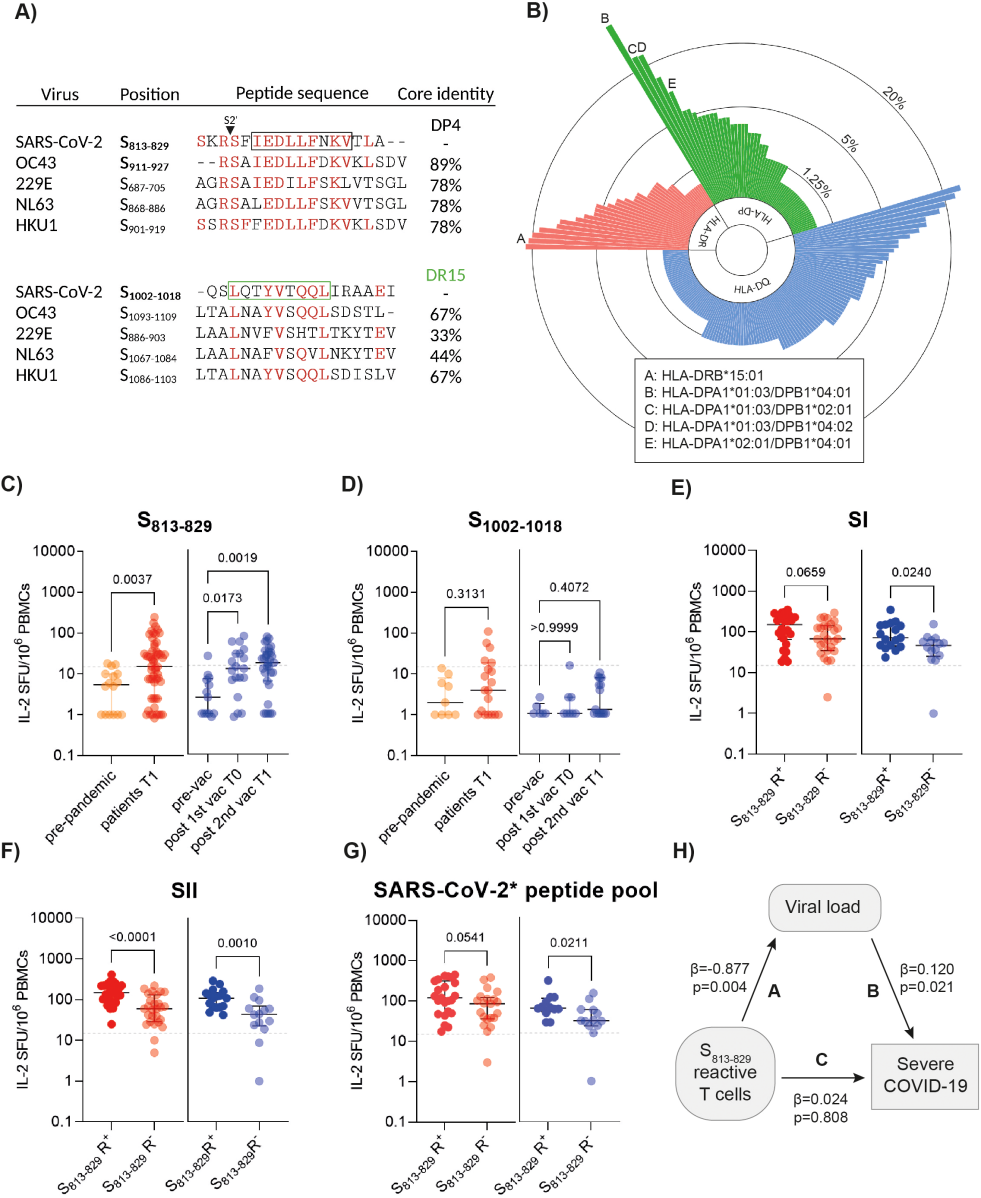
SARS-CoV-2 T cell responses to cross-reactive peptides in patients and vaccinated individuals. **(A)** Sequence identity of common cold coronavirus peptide homologs and SARS-CoV-2-derived peptides in spike S2. Predicted core sequences for HLA-DP4 (black) and HLA-DR15 (green) are indicated by squares. Created with BioRender.com. **(B)** The circular bar plot illustrates the frequency of different HLA-DP, -DR and -DQ alleles (n=222) present in the study population. HLA alleles present at frequencies ≥10% in the study cohort with strong binding affinity for epitopes S_813-829_ and S_1002-1018_ (NetMHCpan% ranks<2) are indicated. **(C)** *Ex vivo* IL-2 ELISpot results after stimulation of PBMCs with spike S_813-829_ in HLA-matched pre-pandemic donors (n=16), patients (n=55) and vaccinated individuals (n=31). **(D)** *Ex vivo* interleukin (IL)-2 ELISpot results after stimulation of PBMCs with spike S_1002-1018_ in HLA-matched unexposed donors (n=9), patients (n=19) and vaccinated individuals (n=17). **(E-G)** *Ex vivo* IL-2 ELISpot results after stimulation of PBMCs with spike SI and SII peptide pools and a pool of non-cross-reactive spike peptides (SARS-CoV-2* peptide pool) in HLA-matched patients (n=55) and vaccinees (n=31) with or without S_813-829_ responses. Each symbol represents one donor. The horizontal lines indicate medians, with error bars illustrating the interquartile range (IQR). The p-values represent the results of unpaired t-tests and one-way ANOVA. Dotted lines indicate 15 SFU/10^6^ PBMCs. SFU, spot forming units. **(H)** Structural equation model illustrating the relationship between S_813-829_-specific T cells, viral load and severe COVID-19. Path A: Multiple regression analysis of S_813-829_-reactive T cells and virus RNA load (standardized regression coefficient ß=-0.877, p=0.004). Path B: The effect of virus RNA load on disease severity (hospitalized vs. non-hospitalized; standardized regression coefficient ß=0.120, p=0.021). Path C: Effect of S_813-829_-specific T cells on severe COVID-19 (standardized regression coefficient ß=0.024, p=0.808). Regression analyses included age as a covariate.

To assess cross-reactivity in SARS-CoV-2 T cell responses, we focused on two previously described, experimentally validated, cross-reactive HLA class II epitopes in the spike S2 domain (44): S_813-829_, presented by HLA-DP4 (HLA-DPA1*01:03/DPB1*04:01; 50% frequency, Europe; 15-80%, world-wide (45–47)), and S_1002-1018_, presented by HLA-DR15 (HLA-DRB*15:01; 13% frequency, Europe; 11% world-wide (46)). The epitopes are conserved between SARS-CoV-2 and CoVs (**Figure 2A**), in particular OC43, with homologous peptides presented by the same HLA-II molecules (35). The HLA-DP4 and HLA-DR15 core sequences differ by one (S_813-829_) or three residues (S_1002-1018_) between SARS-CoV-2 and OC43, respectively (**Figure 2A**). We performed HLA-II genotyping and *in silico* prediction of peptide binding strength via NetMHCpan4.1EL (39). From 222 distinct HLA-II alleles present in our study cohort, we selected a subcohort of participants with HLA-DP4, HLA-DR15 or three additional HLA-DP alleles that met two criteria: (i) predicted with high binding scores to S_813-829_ or S_1002-1018_, with percentile ranks <2 and (ii) detected at frequencies ≥10% in the study population (**Figure 2B**, **Supplementary Table 2**). Consistent with previous findings, we observed T cell reactivity to S_813-829_ in pre-pandemic samples from 3 of 16 HLA-matched blood donors, whereas no significant T cell reactivity was detected against S_1002-1018_ (**Supplementary Figure 1A**). To confirm the S_813-829_ peptide as a CoV-cross-reactive epitope, we analyzed responses against SARS-CoV-2 S_813-829_ or the homologous OC43 S_911-927_ peptide in PBMCs from the three pre-pandemic donors. After 10-day *in vitro* stimulation with SARS-CoV-2 S_813-829_ or the OC43 S_911-927_ peptide, we assessed cytokine production (interferon gamma (IFN-γ), interleukin (IL)-2, and tumor necrosis factor alpha (TNF-α)) by intracellular cytokine staining (ICS) after restimulation with S_813-829_ or the homologous OC43 S_911-927_ peptide. Paired analysis revealed statistically significant differences in IFN-γ and IL-2 responses of SARS-CoV-2 S_813-829_ or OC43 S_911-927_-stimulated samples compared with unstimulated controls in several PBMC lines generated from 2 out of 3 donors (**Supplementary Table 3**). Results from two representative cell lines are illustrated in **Supplementary Figure 1B**. PBMC lines generated with SARS-CoV-2 S_813-829_ showed cross-reactivity with the OC43 S_911-927_ peptide (**Supplementary Figure 1C**). Furthermore, we generated six cell lines following 10-day stimulation with OC43 S_911-927_, and confirmed that these cells were cross-reactive with the SARS-CoV-2 S_813-829_ peptide (**Supplementary Figure 1C**). These data indicate that cross-reactive T cells recognizing the CoV OC43 and SARS-CoV-2 spike epitopes were present in unexposed individuals.

### 3.3 Spike S_813-829_-specific T cell responses correlate with reduced viral load

We next examined *ex vivo* ELISpot responses against S_813-829_ and spike SI and SII peptide pools in a sub-cohort of 55 individuals with SARS-CoV-2 infection and 31 individuals who received BNT162b2 mRNA vaccination and carried the relevant HLA-DP or HLA-DR alleles. While only 15% (5/34) HLA-DR15^+^ individuals displayed a response to the S_1002-1018_ peptide, S_813-829_-reactive T cells were detected in 51% COVID-19 patients and in 55% individuals after the 2^nd^ vaccine dose, with significantly higher frequencies than in non-exposed donors (**Figures 2C, D**). Of note, samples from individuals who possessed S_813-829_-specific T cells (herein referred to as S_813-829_ responders, S_813-829_ R^+^) displayed significantly enhanced SII-specific responses compared with those in whom S_813-829_-specific T cells were not detected (S_813-829_ non-responders, S_813-829_ R^-^) (**Figures 2E, F**). In contrast, less pronounced or no differences were observed between S_813-829_ responders and non-responders when we analyzed T cell reactivity to a pool of 22 SARS-CoV-2 peptides in non-conserved regions of the spike with no homolog in CoVs (SARS-CoV-2* peptide pool; **Supplementary Table 4**). As illustrated in **Figure 2G**, individuals mounted robust SARS-CoV-2* peptide pool responses that were similar or higher in S_813-829_ R^+^ than in S_813-829_ R^-^, suggesting that cross-reactive T cells did not abrogate the formation of *de novo* SARS-CoV-2 T cell responses.

To further examine the impact of cross-reactive T cells, we performed viral RNA (vRNA) load analysis from nasopharyngeal secretions, available from 44 COVID-19 patients (mild infection, n=15; hospitalized with severe disease, n=29). At one month after symptom onset, 40.9% (18/44) of the patients displayed detectable vRNA (range: 4x10^2^-1.6x10^7^ copies/ml). Univariate analyses revealed that lower vRNA loads correlated with higher T cell levels against S_813-829_ (p<0.001; R^2^ = 0.26) and SII peptides (p=0.022; R^2^ = 0.12). By contrast, higher vRNA load, older age and increased reactivity to SI peptides correlated with severe disease (hospitalization) (**Supplementary Tables 5** and **6**). Linear regression analysis revealed an age-dependent decline in S_813-829_ T cell levels (R^2^ = 0.1187, p=0.01), consistent with previous studies (3). Therefore, we performed multiple regression analyses, including age as a covariate, to assess the effect of cross-reactive T cells on vRNA load. This analysis indicated that S_813-829_-specific T cell reactivity correlated with significantly reduced viral loads (p=0.004; regression coefficient -0.877) (**Figure 2H**). When we analyzed the impact of S_813-829_-specific T cells and viral load on disease severity using structural equation modeling with age as a covariate, we observed that while the direct effect of S_813-829_-specific T cells on disease severity was not significant (p=0.808; standardized regression coefficient 0.024), the indirect effect, mediated by viral load reduction was significant (p=0.021; standardized regression coefficient 0.12) (**Figure 2H**).

### 3.4 Cross-reactive T cell immunity contributes to long-lived memory response

**Figure 3:**
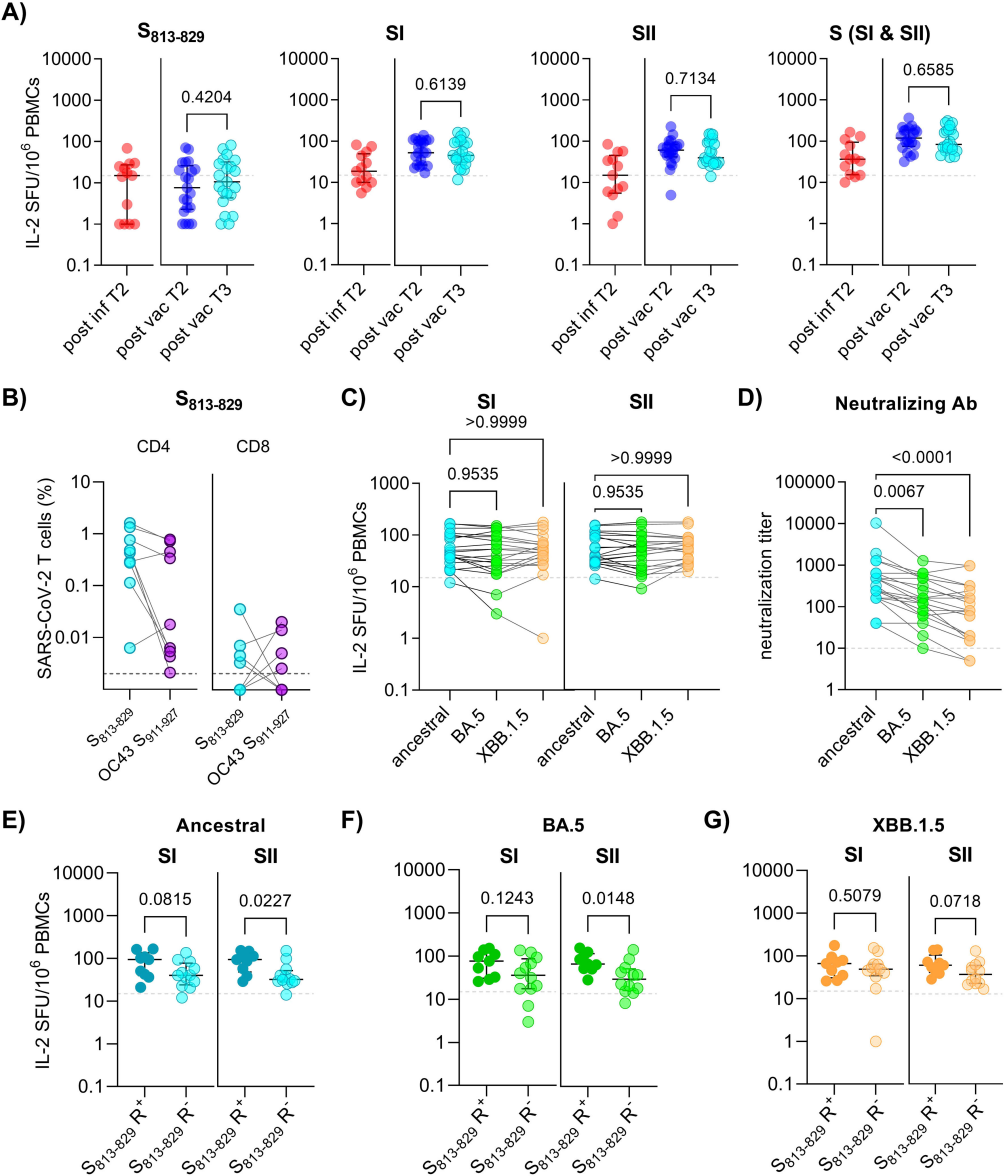
Durability and cross-reactivity of spike-specific T cells in individuals 3 years after first SARS-CoV-2 exposure. **(A)** *Ex vivo* interleukin (IL)-2 ELISpot results after stimulation of PBMCs with spike S_813-829_, SI and SII peptide pools in HLA-matched individuals at 6 months post-infection (post inf T2; n=13), and 1 year (post vac T2; n=21) and 3 years (post vac T3; n=21) post 2^nd^ vaccination, respectively. Each symbol represents one donor. **(B)** PBMCs obtained 3 years post 2^nd^ vaccination were propagated for 10 days with S_813-829,_ followed by flow cytometric analysis after restimulation with indicated peptides. Graphs show frequencies of IL-2, IFN-γ and TNF-α-positive cells within CD4 (left panel) and CD8 (right panel) (n=9; shown for S_813-829_ R^+^). **(C)** *Ex vivo* IL-2 ELISpot results after stimulation with spike SI and SII peptide pools covering the ancestral and omicron variants BA.5 and XBB.1.5 in HLA-matched individuals 3 years post-vaccination. **(D)** Serum neutralization titers against ancestral and omicron BA.5 and XBB.1.5 variants at 3 years post-vaccination. **(E-G)** *Ex vivo* IL-2 ELISpot results after stimulation of PBMCs with spike SI and SII peptide pools covering the ancestral or omicron BA.5 and XBB.1.5 variants in HLA-matched donors (n=21) with or without S_813-829_ responses (S_813-829_ R^+^ and S_813-829_ R^-^, respectively). The horizontal lines indicate medians, with error bars illustrating the interquartile range (IQR). The p-values represent the results of unpaired t-tests **(A, E-G)**, and Dunn´s multiple comparisons tests **(C,D)**. Dotted lines indicate 15 SFU/10^6^ PBMCs. SFU, spot forming units.

A key unresolved question is whether CoV-cross-reactive T cells contribute to immune responses beyond the first SARS-CoV-2 encounter. To measure T cell maintenance, we compared IL-2^+^ T cell frequencies to S_813-829_ and spike SI and SII peptide pools in paired samples 6-12 months apart (**Figure 1A**). Follow-up samples from patients were taken 6 months post infection (T2, n=13), and from vaccinated individuals one year after 2^nd^ vaccination (T2, n=21); all vaccinated individuals had received a booster dose at a median of 3 months before sample collection (**Supplementary Table 1**). A significant reduction in IL-2^+^ T cell frequencies to S_813-829_ and SI and SII peptide pools was observed between acute SARS-CoV-2 infection and 6 months post-infection, while IL-2^+^ T cell levels in vaccinated individuals who had received a booster dose were not different to those post 2^nd^ vaccination (**Supplementary Figure 2**). Notably, S_813-829_-specific T cells could still be detected in a significant proportion of individuals both after infection (46%) or vaccination (38%) (**Figure 3A**). To assess long-term T cell persistence, we compared IL-2^+^ T cell frequencies to S_813-829_ and spike SI and SII peptide pools after booster vaccination with follow-up samples obtained approximately 3 years post 2^nd^ vaccination (T3, median: 2.5 years after booster vaccination) (**Supplementary Table 1**). Between time points T2 and T3, 76% (16/21) of the individuals received one to three additional booster doses, including bivalent wild-type/BA.5 vaccine (n = 7) and/or monovalent XBB.1.5 vaccine (n = 9), and 71% (15/21) had a confirmed omicron breakthrough infection (**Supplementary Table 7**). Evaluations of IL-2^+^ T cell frequencies showed no significant differences in the response against spike SI or SII and S_813-829_ between T2 and T3 (**Figure 3A**). Since T cell responses to S_813-829_ were maintained in three-year-follow-up samples, we analyzed the frequency of these cells targeting the CoV OC43 homologous peptide. PBMCs were propagated with S_813-829_ for 10 days and restimulated with S_813-829_ or the OC43 S_911-927_ peptide. Responsive cells were identified by expression of IFN-γ, IL-2 or TNF-α production by flow cytometry. Despite substantial individual variability in the frequencies of responses, we detected CD4 T cells targeting SARS-CoV-2 S_813-829_ and OC43 S_911-927_ peptides in all individuals who mounted S_813-829_-specific responses, while the proportions of CD8 T cells targeting these peptides were considerably lower (**Figure 3B**). Analysis of cytokine-positive subsets, including polyfunctional cells (defined as cells producing ≥ 2 cytokines) and single cytokine positive cells revealed that SARS-CoV-2 S_813-829_ and OC43 S_911-927_ peptides induced mainly triple- and dual-positive cells with positive correlations between individual IL-2 and IFN-γ subsets (Spearman R=-0.81, p<0.0001), and IL-2 and TNF-α subsets (Spearman R=-0.98, p<0.0001) (**Supplementary Figure 3**). In summary, these data indicate that SARS-CoV-2 S_813-829_ peptide induced polyfunctional cross-reactive CD4 T cell populations, and that a substantial proportion of these cells was maintained in the memory T cell pool.

Furthermore, we compared the frequency of T cells targeting ancestral and omicron BA.5 and XBB.1.5 spike peptides. The data revealed similar frequencies of spike SI and SII T cell responses across these variants (**Figure 3C**). In contrast, serum samples from these individuals showed significantly reduced neutralizing antibody titers against omicron BA.5 and XBB.1.5 variants compared to the ancestral strain, demonstrating a significant escape from antibody-mediated neutralization (**Figure 3D**).

To assess whether cross-reactive T cells were associated with enhanced T cell responses to Omicron variants, we compared spike-specific responses between S_813-829_ R+ and S_813-829_ R-. The analysis revealed that S_813-829_ responders displayed significantly enhanced SII-specific T cell responses to ancestral and omicron BA.5 spike compared with those in whom S_813-829_-specific T cells were not detected (ancestral, p=0.0227; BA.5, p=0.0148) (**Figures 3E, F**). For omicron XBB.1.5, a similar trend for enhanced SII-specific T cell levels in S_813-829_ R^+^ vs S_813-829_ R^-^ was observed, although the difference was not statistically significant (p=0.0718) (**Figure 3G**). Together, the data suggest that S_813-829_ contributes to long-lasting memory T cells that recognize CoV OC43 and SARS-CoV-2 variants, such as omicron BA.5 and XBB.1.5.

## 4 Discussion

Increasing evidence suggests that cross-reactive T cell immunity influences COVID-19 vaccine responses and disease outcome (1, 3, 6, 22, 27, 30, 31, 48–50), yet we still lack an understanding of its effects beyond an individual´s first SARS-CoV-2 encounter. We characterized spike cross-reactive T cell immunity, its association with infection outcomes, COVID-19 severity, and its role in durable immune responses in HLA-matched cohorts from 2020 to 2024. In particular, our data show that CoV-reactive and SARS-CoV-2 cross-reactive T cells account for significantly enhanced and sustained spike-specific T cell responses in individuals after SARS-CoV-2 vaccination and infection. We identified a highly conserved peptide within the coronavirus spike fusion domain (S_813-829_), which showed higher responder rates than the less conserved S_1002-1018_ peptide, consistent with previous studies (51). Notably, the S_813-829_ peptide overlaps significantly with recently described immunodominant sequences (3, 5, 21, 44, 52, 53) shown to enhance responsiveness to both SARS-CoV-2 infection and vaccination (3). We observed that these responses are sustained over time, with spike cross-reactive T cell immunity correlating with enhanced responses against SARS-CoV-2 ancestral strain and highly mutated omicron variants. At the same time, we found that the magnitude of T cell responses against the N-terminal part of the spike (SI peptide pool) was similar in individuals with or without S_813-829_-specific T cells, suggesting that the individual propensity to generate CD4 T cell responses was comparable between these groups.

Given that the S_813-829_ sequence is entirely conserved across SARS-CoV-2 strains, it is likely that T cell responses will be recalled upon infection with SARS-CoV-2 variants. In addition, we observed that S_813-829_-propagated T cell lines from almost all individuals mounted cross-reactivity with CoV OC43. Thus, our finding of a sustained cross-reactive memory T cell pool during a three-year follow-up period may reflect the persistence of long-lived memory T cells (20, 53–55) as well as repeated exposure to SARS-CoV-2 variants or CoV reinfections, boosting cross-reactive T cells. Remarkably, we observed a high degree of T cell cross-reactivity across different SARS-CoV-2 variants, which is supported by recent studies (56–61), and contrasts with the reduced neutralizing capacity of serum antibodies in these individuals, even in those who had two or more exposures to omicron spike protein due to breakthrough infection and/or omicron vaccination.

Our study highlights the impact of coronavirus-spike cross-reactive T cells, which was evident in stronger and long-lasting T cell responses. Furthermore, multiple regression analysis, including age as a co-variate, indicated that cross-reactive T cells were associated with a more rapid control of virus replication. Furthermore, multivariate analyses revealed no additional correlation between S_813-829_-specific T cells and disease severity after adjustment for vRNA load, consistent with the notion that T cells might affect clinical outcomes mainly by limiting viral replication. Analysis of *in vitro* S_813-829_-propagated T cell lines showed that these responses were driven primarily by polyfunctional CD4 T cells. These cells likely contribute to viral control by enhancing cytotoxic T cell activity and direct cytotoxicity (62–64). Recent evidence from mouse studies supports a protective role of cross-reactive CD4 T cells against SARS-CoV-2 following OC43 infection, with the level of protection varying significantly depending on the specific HLA allele (28). The importance of HLA types with respect to cross-reactive T cells has also been demonstrated by studies in COVID-19 patients, showing that HLA genotype is a key determinant both in the establishment and recall of pre-existing SARS-CoV-2 T cells (32). In this context, it should be noted that our study on cross-reactive T cell responses, including pre-exposure T cell frequencies, disease outcomes, and long-lasting T cell memory was conducted with a focus on 4 common HLA-II alleles (44), particularly DP4 (DP401 and DP402), which are among the most prevalent alleles worldwide (65). The broad distribution of HLA molecules presenting S_813-829_, combined with its conservation across human seasonal coronaviruses, makes it a stable and reliable target for sustained and lasting immune memory responses across diverse populations, potentially mitigating the impact of future SARS-CoV-2 variants or newly emerging coronaviruses.

A limitation of our study is that we did not use ELISpot assays to assess ex vivo T cell responses against the OC43 S_911-927_ peptide, which in this study were identified using ICS staining after 10-day *in vitro* peptide stimulation. An additional limitation is that our cohort of pre-pandemic donors was small and we did not investigate further details of the peptide-expanded T cells due to limited blood volumes. Furthermore, data on the details of patient comorbidities could not be retrieved from our anonymized samples, and the combined effects of age and comorbidities on T cell responses and clinical outcomes will have to be resolved in future clinical studies. Lastly, our approach may have missed T cell responses not associated with IL-2 production or those below the detection threshold of ex vivo ELISPOT assays.

In summary, our findings highlight the impact of T cell cross-reactivity, demonstrating that it contributes both to initial SARS-CoV-2 immune responses and to the durability of memory responses. The effects observed include an increased spike T cell response that correlates with more rapid viral control as well as durably enhanced memory T cells, capable of recognizing CoV and highly mutated omicron variants.

## Data Availability

The datasets presented in this study can be found in online repositories. The names of the repository/repositories and accession number(s) can be found below: EPI_ISL_18638453 (GISAID; https://www.epicov.org/epi3/frontend#4c7371).

## References

[1] Kundu R Narean JS Wang L Fenn J Pillay T Fernandez ND. Cross-reactive memory T cells associate with protection against SARS-CoV-2 infection in COVID-19 contacts. Nat Commun. (2022) 13:80. doi: 10.1038/s41467-021-27674-x 35013199 PMC8748880

[2] Swadling L Diniz MO Schmidt NM Amin OE Chandran A Shaw E. Pre-existing polymerase-specific T cells expand in abortive seronegative SARS-CoV-2. Nature. (2022) 601:110–7. doi: 10.1038/s41586-021-04186-8 PMC8732273 34758478

[3] Loyal L Braun J Henze L Kruse B Dingeldey M Reimer U. Cross-reactive CD4(+) T cells enhance SARS-CoV-2 immune responses upon infection and vaccination. Science. (2021) 374:eabh1823. doi: 10.1126/science.abh1823 34465633 PMC10026850

[4] Chandran A Rosenheim J Nageswaran G Swadling L Pollara G Gupta RK. Rapid synchronous type 1 IFN and virus-specific T cell responses characterize first wave non-severe SARS-CoV-2 infections. Cell Rep Med. (2022) 3:100557. doi: 10.1016/j.xcrm.2022.100557 35474751 PMC8895494

[5] Tan AT Linster M Tan CW Le Bert N Chia WN Kunasegaran K. Early induction of functional SARS-CoV-2-specific T cells associates with rapid viral clearance and mild disease in COVID-19 patients. Cell Rep. (2021) 34:108728. doi: 10.1016/j.celrep.2021.108728 33516277 PMC7826084

[6] Mateus J Dan JM Zhang Z Rydyznski Moderbacher C Lammers M Goodwin B. Low-dose mRNA-1273 COVID-19 vaccine generates durable memory enhanced by cross-reactive T cells. Science. (2021) 374:eabj9853. doi: 10.1126/science.abj9853 34519540 PMC8542617

[7] Israel A Shenhar Y Green I Merzon E Golan-Cohen A Schaffer AA. Large-Scale Study of Antibody Titer Decay following BNT162b2 mRNA Vaccine or SARS-CoV-2 Infection. Vaccines (Basel). (2021) 10. doi: 10.3390/vaccines10010064 PMC8781423 35062724

[8] Cao Y Yisimayi A Jian F Song W Xiao T Wang L. BA.2.12.1, BA.4 and BA.5 escape antibodies elicited by Omicron infection. Nature. (2022) 608:593–602. doi: 10.1038/s41586-022-04980-y 35714668 PMC9385493

[9] Khan K Karim F Ganga Y Bernstein M Jule Z Reedoy K. Omicron BA.4/BA.5 escape neutralizing immunity elicited by BA.1 infection. Nat Commun. (2022) 13:4686. doi: 10.1038/s41467-022-32396-9 35948557 PMC9364294

[10] Springer DN Bauer M Medits I Camp JV Aberle SW Burtscher C. Bivalent COVID-19 mRNA booster vaccination (BA.1 or BA.4/BA.5) increases neutralization of matched Omicron variants. NPJ Vaccines. (2023) 8:110. doi: 10.1038/s41541-023-00708-9 37542025 PMC10403593

[11] Springer DN Medits I Weseslindtner L Stiasny K Aberle JH. SARS-CoV-2 neutralising antibody response to bivalent booster after omicron infection. Lancet Microbe. (2024) 5:e8. doi: 10.1016/S2666-5247(23)00293-8 37918419

[12] Liu J Yu J McMahan K Jacob-Dolan C He X Giffin V. CD8 T cells contribute to vaccine protection against SARS-CoV-2 in macaques. Sci Immunol. (2022) 7:eabq7647. doi: 10.1126/sciimmunol.abq7647 35943359 PMC9407944

[13] Kedzierska K Thomas PG. Count on us: T cells in SARS-CoV-2 infection and vaccination. Cell Rep Med. (2022) 3:100562. doi: 10.1016/j.xcrm.2022.100562 35474748 PMC8872824

[14] Springer DN Camp JV Aberle SW Deutsch J Lammel O Weseslindtner L. Neutralization of SARS-CoV-2 Omicron XBB.1.5 and JN.1 variants after COVID-19 booster-vaccination and infection. J Med Virol. (2024) 96:e29801. doi: 10.1002/jmv.29801 38988204

[15] Cromer D Juno JA Khoury D Reynaldi A Wheatley AK Kent SJ. Prospects for durable immune control of SARS-CoV-2 and prevention of reinfection. Nat Rev Immunol. (2021) 21:395–404. doi: 10.1038/s41577-021-00550-x 33927374 PMC8082486

[16] Guo L Wang G Wang Y Zhang Q Ren L Gu X. SARS-CoV-2-specific antibody and T-cell responses 1 year after infection in people recovered from COVID-19: a longitudinal cohort study. Lancet Microbe. (2022) 3:e348–e56. doi: 10.1016/S2666-5247(22)00036-2 PMC8942480 35345417

[17] Feng C Shi J Fan Q Wang Y Huang H Chen F. Protective humoral and cellular immune responses to SARS-CoV-2 persist up to 1 year after recovery. Nat Commun. (2021) 12:4984. doi: 10.1038/s41467-021-25312-0 34404803 PMC8370972

[18] Jung JH Rha MS Sa M Choi HK Jeon JH Seok H. SARS-CoV-2-specific T cell memory is sustained in COVID-19 convalescent patients for 10 months with successful development of stem cell-like memory T cells. Nat Commun. (2021) 12:4043. doi: 10.1038/s41467-021-24377-1 34193870 PMC8245549

[19] Dan JM Mateus J Kato Y Hastie KM Yu ED Faliti CE. Immunological memory to SARS-CoV-2 assessed for up to 8 months after infection. Science. (2021) 371(6529):eabf4063. doi: 10.1126/science.abf4063 33408181 PMC7919858

[20] Le Bert N Tan AT Kunasegaran K Tham CYL Hafezi M Chia A. SARS-CoV-2-specific T cell immunity in cases of COVID-19 and SARS, and uninfected controls. Nature. (2020) 584:457–62. doi: 10.1038/s41586-020-2550-z 32668444

[21] Mateus J Grifoni A Tarke A Sidney J Ramirez SI Dan JM. Selective and cross-reactive SARS-CoV-2 T cell epitopes in unexposed humans. Science. (2020) 370:89–94. doi: 10.1126/science.abd3871 32753554 PMC7574914

[22] Braun J Loyal L Frentsch M Wendisch D Georg P Kurth F. SARS-CoV-2-reactive T cells in healthy donors and patients with COVID-19. Nature. (2020) 587:270–4. doi: 10.1038/s41586-020-2598-9 32726801

[23] Murray SM Ansari AM Frater J Klenerman P Dunachie S Barnes E. The impact of pre-existing cross-reactive immunity on SARS-CoV-2 infection and vaccine responses. Nat Rev Immunol. (2023) 23:304–16. doi: 10.1038/s41577-022-00809-x PMC9765363 36539527

[24] Jay C Adland E Csala A Dold C Edmans M Hackstein CP. Cellular immunity to SARS-CoV-2 following intrafamilial exposure in seronegative family members. Front Immunol. (2023) 14:1248658. doi: 10.3389/fimmu.2023.1248658 37711627 PMC10497976

[25] Ogbe A Kronsteiner B Skelly DT Pace M Brown A Adland E. T cell assays differentiate clinical and subclinical SARS-CoV-2 infections from cross-reactive antiviral responses. Nat Commun. (2021) 12:2055. doi: 10.1038/s41467-021-21856-3 33824342 PMC8024333

[26] Meyerholz DK Perlman S. Does common cold coronavirus infection protect against severe SARS-CoV-2 disease? J Clin Invest. (2021) 131(1):e144807. doi: 10.1172/JCI144807 33216734 PMC7773392

[27] Sagar M Reifler K Rossi M Miller NS Sinha P White LF. Recent endemic coronavirus infection is associated with less-severe COVID-19. J Clin Invest. (2021) 131(1):e143380. doi: 10.1172/JCI143380 32997649 PMC7773342

[28] Dos Santos Alves RP Timis J Miller R Valentine K Pinto PBA Gonzalez A. Human coronavirus OC43-elicited CD4(+) T cells protect against SARS-CoV-2 in HLA transgenic mice. Nat Commun. (2024) 15:787. doi: 10.1038/s41467-024-45043-2 38278784 PMC10817949

[29] Saletti G Gerlach T Jansen JM Molle A Elbahesh H Ludlow M. Older adults lack SARS CoV-2 cross-reactive T lymphocytes directed to human coronaviruses OC43 and NL63. Sci Rep. (2020) 10:21447. doi: 10.1038/s41598-020-78506-9 33293664 PMC7722724

[30] Mallajosyula V Ganjavi C Chakraborty S McSween AM Pavlovitch-Bedzyk AJ Wilhelmy J. CD8(+) T cells specific for conserved coronavirus epitopes correlate with milder disease in COVID-19 patients. Sci Immunol. (2021) 6(61):eabg5669. doi: 10.1126/sciimmunol.abg5669 34210785 PMC8975171

[31] Bonifacius A Tischer-Zimmermann S Dragon AC Gussarow D Vogel A Krettek U. COVID-19 immune signatures reveal stable antiviral T cell function despite declining humoral responses. Immunity. (2021) 54:340–54 e6. doi: 10.1016/j.immuni.2021.01.008 33567252 PMC7871825

[32] Francis JM Leistritz-Edwards D Dunn A Tarr C Lehman J Dempsey C. Allelic variation in class I HLA determines CD8(+) T cell repertoire shape and cross-reactive memory responses to SARS-CoV-2. Sci Immunol. (2022) 7:eabk3070. doi: 10.1126/sciimmunol.abk3070 34793243 PMC9017864

[33] Sette A Sidney J Crotty S. T cell responses to SARS-coV-2. Annu Rev Immunol. (2023) 41:343–73. doi: 10.1146/annurev-immunol-101721-061120 36750314

[34] Popa A Genger JW Nicholson MD Penz T Schmid D Aberle SW. Genomic epidemiology of superspreading events in Austria reveals mutational dynamics and transmission properties of SARS-CoV-2. Sci Transl Med. (2020) 12(573):eabe2555. doi: 10.1126/scitranslmed.abe2555 33229462 PMC7857414

[35] Becerra-Artiles A Nanaware PP Muneeruddin K Weaver GC Shaffer SA Calvo-Calle JM. Immunopeptidome profiling of human coronavirus OC43-infected cells identifies CD4 T-cell epitopes specific to seasonal coronaviruses or cross-reactive with SARS-CoV-2. PLoS Pathog. (2023) 19(7):e1011032. doi: 10.1371/journal.ppat.1011032 37498934 PMC10409285

[36] Corman VM Landt O Kaiser M Molenkamp R Meijer A Chu DKW. Detection of 2019 novel coronavirus (2019-nCoV) by real-time RT-PCR. Eurosurveillance. (2020) 25:23–30. doi: 10.2807/1560-7917.ES.2020.25.3.2000045 PMC6988269 31992387

[37] Fae I Wenda S Grill C Fischer GF. HLA-B*44:138Q: Evidence for a confined deletion and recombination events in an otherwise unaffected HLA-haplotype. Hla. (2019) 93:89–96. doi: 10.1111/tan.13439 30488584 PMC6590401

[38] Koblischke M Spitzer FS Florian DM Aberle SW Malafa S Fae I. CD4 T cell determinants in west nile virus disease and asymptomatic infection. Front Immunol. (2020) 11:16. doi: 10.3389/fimmu.2020.00016 32038660 PMC6989424

[39] Nilsson JB Kaabinejadian S Yari H Kester MGD van Balen P Hildebrand WH. Accurate prediction of HLA class II antigen presentation across all loci using tailored data acquisition and refined machine learning. Sci Adv. (2023) 9:eadj6367. doi: 10.1126/sciadv.adj6367 38000035 PMC10672173

[40] Koblischke M Mackroth MS Schwaiger J Fae I Fischer G Stiasny K. Protein structure shapes immunodominance in the CD4 T cell response to yellow fever vaccination. Sci Rep-Uk. (2017) 7(1):8907. doi: 10.1038/s41598-017-09331-w PMC5566484 28827760

[41] Koblischke M Traugott MT Medits I Spitzer FS Zoufaly A Weseslindtner L. Dynamics of CD4 T cell and antibody responses in COVID-19 patients with different disease severity. Front Med (Lausanne). (2020) 7:592629. doi: 10.3389/fmed.2020.592629 33262993 PMC7686651

[42] Agerer B Koblischke M Gudipati V Montano-Gutierrez LF Smyth M Popa A. SARS-CoV-2 mutations in MHC-I-restricted epitopes evade CD8(+) T cell responses. Sci Immunol. (2021) 6(57):eabg6461. doi: 10.1126/sciimmunol.abg6461 33664060 PMC8224398

[43] Hollstein MM Dierks S Schon MP Bergmann A Abratis A Eidizadeh A. Humoral and cellular immune responses in fully vaccinated individuals with or without SARS-CoV-2 breakthrough infection: Results from the CoV-ADAPT cohort. J Med Virol. (2023) 95:e29122. doi: 10.1002/jmv.v95.10 37787583

[44] Becerra-Artiles A Calvo-Calle JM Co MD Nanaware PP Cruz J Weaver GC. Broadly recognized, cross-reactive SARS-CoV-2 CD4 T cell epitopes are highly conserved across human coronaviruses and presented by common HLA alleles. Cell Rep. (2022) 39:110952. doi: 10.1016/j.celrep.2022.110952 35675811 PMC9135679

[45] Castelli FA Buhot C Sanson A Zarour H Pouvelle-Moratille S Nonn C. HLA-DP4, the most frequent HLA II molecule, defines a new supertype of peptide-binding specificity. J Immunol. (2002) 169:6928–34. doi: 10.4049/jimmunol.169.12.6928 12471126

[46] Gonzalez-Galarza FF McCabe A Santos E Jones J Takeshita L Ortega-Rivera ND. Allele frequency net database (AFND) 2020 update: gold-standard data classification, open access genotype data and new query tools. Nucleic Acids Res. (2020) 48:D783–D8. doi: 10.1093/nar/gkz1029 PMC7145554 31722398

[47] Sidney J Steen A Moore C Ngo S Chung J Peters B. Five HLA-DP molecules frequently expressed in the worldwide human population share a common HLA supertypic binding specificity. J Immunol. (2010) 184:2492–503. doi: 10.4049/jimmunol.0903655 PMC2935290 20139279

[48] Grifoni A Weiskopf D Ramirez SI Mateus J Dan JM Moderbacher CR. Targets of T cell responses to SARS-coV-2 coronavirus in humans with COVID-19 disease and unexposed individuals. Cell. (2020) 181:1489–501 e15. doi: 10.1016/j.cell.2020.05.015 32473127 PMC7237901

[49] Schulien I Kemming J Oberhardt V Wild K Seidel LM Killmer S. Characterization of pre-existing and induced SARS-CoV-2-specific CD8(+) T cells. Nat Med. (2021) 27:78–85. doi: 10.1038/s41591-020-01143-2 33184509

[50] Le Bert N Clapham HE Tan AT Chia WN Tham CYL Lim JM. Highly functional virus-specific cellular immune response in asymptomatic SARS-CoV-2 infection. J Exp Med. (2021) 218(5):e20202617. doi: 10.1084/jem.20202617 33646265 PMC7927662

[51] Tarke A Sidney J Kidd CK Dan JM Ramirez SI Yu ED. Comprehensive analysis of T cell immunodominance and immunoprevalence of SARS-CoV-2 epitopes in COVID-19 cases. Cell Rep Med. (2021) 2:100204. doi: 10.1016/j.xcrm.2021.100204 33521695 PMC7837622

[52] Dykema AG Zhang B Woldemeskel BA Garliss CC Cheung LS Choudhury D. Functional characterization of CD4+ T cell receptors crossreactive for SARS-CoV-2 and endemic coronaviruses. J Clin Invest. (2021) 131(10):e146922. doi: 10.1172/JCI146922 33830946 PMC8121515

[53] Low JS Vaqueirinho D Mele F Foglierini M Jerak J Perotti M. Clonal analysis of immunodominance and cross-reactivity of the CD4 T cell response to SARS-CoV-2. Science. (2021) 372:1336–41. doi: 10.1126/science.abg8985 PMC8168615 34006597

[54] Ng OW Chia A Tan AT Jadi RS Leong HN Bertoletti A. Memory T cell responses targeting the SARS coronavirus persist up to 11 years post-infection. Vaccine. (2016) 34:2008–14. doi: 10.1016/j.vaccine.2016.02.063 PMC7115611 26954467

[55] Yu ED Narowski TM Wang E Garrigan E Mateus J Frazier A. Immunological memory to common cold coronaviruses assessed longitudinally over a three-year period pre-COVID19 pandemic. Cell Host Microbe. (2022) 30:1269–78 e4. doi: 10.1016/j.chom.2022.07.012 35932763 PMC9296686

[56] Nesamari R Omondi MA Baguma R Hoft MA Ngomti A Nkayi AA. Post-pandemic memory T cell response to SARS-CoV-2 is durable, broadly targeted, and cross-reactive to the hypermutated BA.2.86 variant. Cell Host Microbe. (2024) 32:162–9 e3. doi: 10.1016/j.chom.2023.12.003 38211583 PMC10901529

[57] Keeton R Tincho MB Ngomti A Baguma R Benede N Suzuki A. T cell responses to SARS-CoV-2 spike cross-recognize Omicron. Nature. (2022) 603:488–92. doi: 10.1038/s41586-022-04460-3 PMC8930768 35102311

[58] Tarke A Coelho CH Zhang Z Dan JM Yu ED Methot N. SARS-CoV-2 vaccination induces immunological T cell memory able to cross-recognize variants from Alpha to Omicron. Cell. (2022) 185:847–59 e11. doi: 10.1016/j.cell.2022.01.015 35139340 PMC8784649

[59] Gao Y Cai C Grifoni A Muller TR Niessl J Olofsson A. Ancestral SARS-CoV-2-specific T cells cross-recognize the Omicron variant. Nat Med. (2022) 28:472–6. doi: 10.1038/s41591-022-01700-x PMC8938268 35042228

[60] Naranbhai V Nathan A Kaseke C Berrios C Khatri A Choi S. T cell reactivity to the SARS-CoV-2 Omicron variant is preserved in most but not all individuals. Cell. (2022) 185:1041–51 e6. doi: 10.1016/j.cell.2022.01.029 35202566 PMC8810349

[61] Riou C Keeton R Moyo-Gwete T Hermanus T Kgagudi P Baguma R. Escape from recognition of SARS-CoV-2 variant spike epitopes but overall preservation of T cell immunity. Sci Transl Med. (2022) 14:eabj6824. doi: 10.1126/scitranslmed.abj6824 34931886 PMC9434381

[62] Moss P. The T cell immune response against SARS-CoV-2. Nat Immunol. (2022) 23:186–93. doi: 10.1038/s41590-021-01122-w 35105982

[63] Kaneko N Boucau J Kuo H-H Perugino C Mahajan VS Farmer JR. Temporal changes in T cell subsets and expansion of cytotoxic CD4+ T cells in the lungs in severe COVID-19. Clin Immunol. (2022) 237:108991. doi: 10.1016/j.clim.2022.108991 35364330 PMC8961941

[64] Meckiff BJ Ramírez-Suástegui C Fajardo V Chee SJ Kusnadi A Simon H. Imbalance of regulatory and cytotoxic SARS-coV-2-reactive CD4+ T cells in COVID-19. Cell. (2020) 183:1340–53.e16. doi: 10.1016/j.cell.2020.10.001 33096020 PMC7534589

[65] Augusto DG Murdolo LD Chatzileontiadou DSM Sabatino JJ Jr. Yusufali T Peyser ND. A common allele of HLA is associated with asymptomatic SARS-CoV-2 infection. Nature. (2023) 620:128–36. doi: 10.1038/s41586-023-06331-x PMC10396966 37468623

