# Supplementary material for: Enhanced and long-lasting SARS-CoV-2 immune memory in individuals with common cold coronavirus cross-reactive T cell immunity
Source: Front Immunol. 2025 Mar 21;16:1501704. doi: 10.3389/fimmu.2025.1501704 (PMC11968687; doi:10.3389/fimmu.2025.1501704)
Supplement: Supplementary file 1 [file DataSheet1.pdf]

Supplementary Material

Enhanced and long-lasting SARS-CoV-2 immune memory in individuals with common cold coronavirus cross-reactive T cell immunity

David M. Florian[1], Michael Bauer[1], Amelie Popovitsch[1], Ingrid Fae[2], David N. Springer[1], Marianne Graninger[1], Marianna Traugott[3], Lukas Weseslindtner[1], Stephan W. Aberle[1], Gottfried Fischer[2], Michael Kundi[4], Karin Stiasny[1], Alexander Zoufaly[3,5], Samuel J. Landry[6] and Judith H. Aberle[1*]

[1]Center for Virology, Medical University of Vienna, Vienna, Austria.

[2]Department of Transfusion Medicine and Cell Therapy, Medical University of Vienna, Austria.

[3]Department of Medicine IV, Klinik Favoriten, Vienna, Austria.

[4]Center for Public Health, Department for Environmental Health, Medical University of Vienna, Vienna, Austria.

[5]Faculty of Medicine, Sigmund Freud University, Vienna, Austria.

[6]Department of Biochemistry and Molecular Biology, Tulane University School of Medicine, New Orleans, Louisiana 70112, United States

*** Correspondence:**
Judith H. Aberle
judith.aberle@meduniwien.ac.at

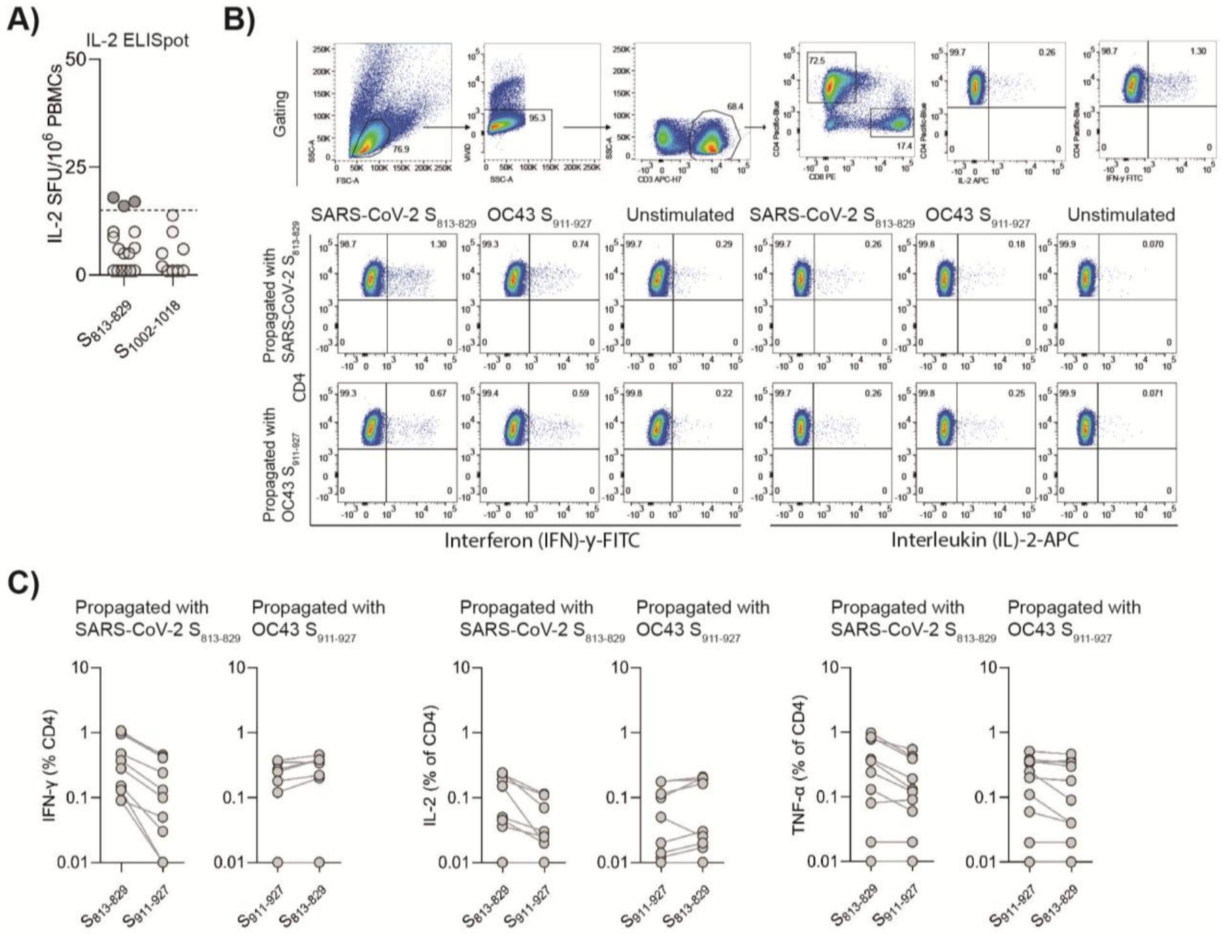

Supplementary Figure 1. SARS-CoV-2 T cell reactivity in pre-pandemic samples from healthy donors. (A) *Ex vivo* interleukin (IL)-2 ELISpot results after stimulation of PBMCs with SARS-CoV-2 spike $S_{813-829}$ or $S_{1002-1018}$ in HLA-matched pre-pandemic donors. Each symbol represents one donor. Filled circles identify donors with responses >15 SFU/10^6 PBMCs. **(B)** PBMCs from three HLA-DP4+ or HLA-DPA1*01:03/04:02+ pre-pandemic donors were propagated for 10 days with indicated peptides and assessed for cytokine production (interferon gamma (IFN-γ), IL-2, and tumor necrosis factor alpha (TNF-α) by intracellular cytokine staining after restimulation with SARS-CoV-2 $S_{813-829}$ and OC43 $S_{911-927}$ peptides. The gating strategy and representative FACS plots for IFN-γ and IL-2 are shown. **(C)** Cytokine response of T cells following 10-day stimulation with SARS-CoV-2 $S_{813-829}$ or OC43 $S_{911-927}$ peptides and restimulation with SARS-CoV-2 $S_{813-829}$ compared to OC43 $S_{911-927}$ peptides. Data presented are after background subtraction (from the frequency of cytokines in unstimulated wells). Two to three lines were evaluated per donor and peptide.

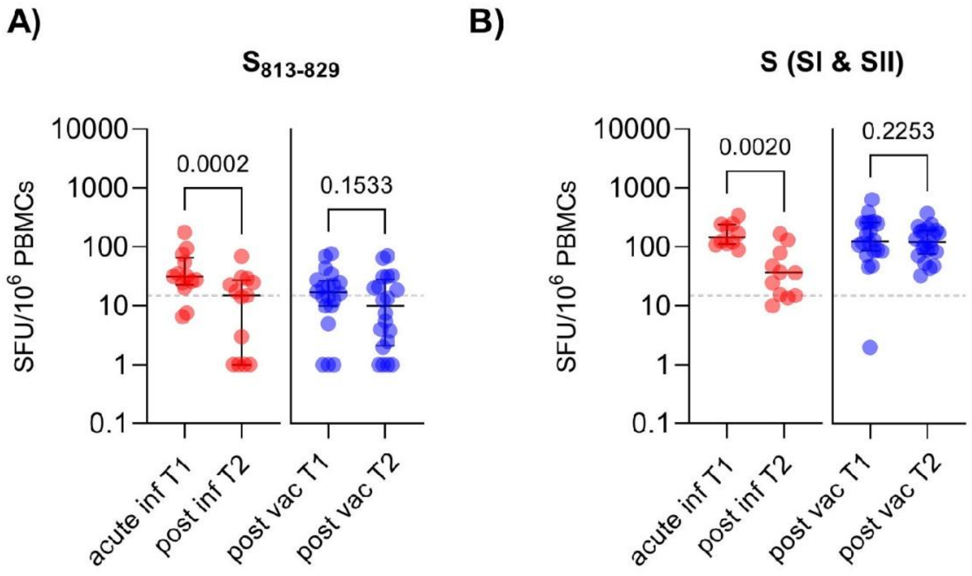

Supplementary Figure 2. Longitudinal analysis of SARS-CoV-2-specific T cells in infected and vaccinated individuals. (A and B) Ex vivo interleukin (IL)-2 (ELISpot results after stimulation of PBMCs with spike $S_{813-829}$ **(A)** and SI and SII peptide pools **(B)**. Paired samples during acute infection (acute inf T1) and at 6 months post infection (post inf T2) (red, n=13), and 2-3 weeks (post vac T1) and 1 year post 2nd vaccination (post vac T2) (blue, n=21); vaccinated individuals received a booster vaccination 3 months before sample collection. Each symbol represents one donor. The horizontal lines indicate median, with error bars illustrating the interquartile range (IQR). The p-values represent the results of Wilcoxon matched-pairs signed rank test. Dotted lines indicate 15 SFU/106 PBMCs. SFU, spot forming units.

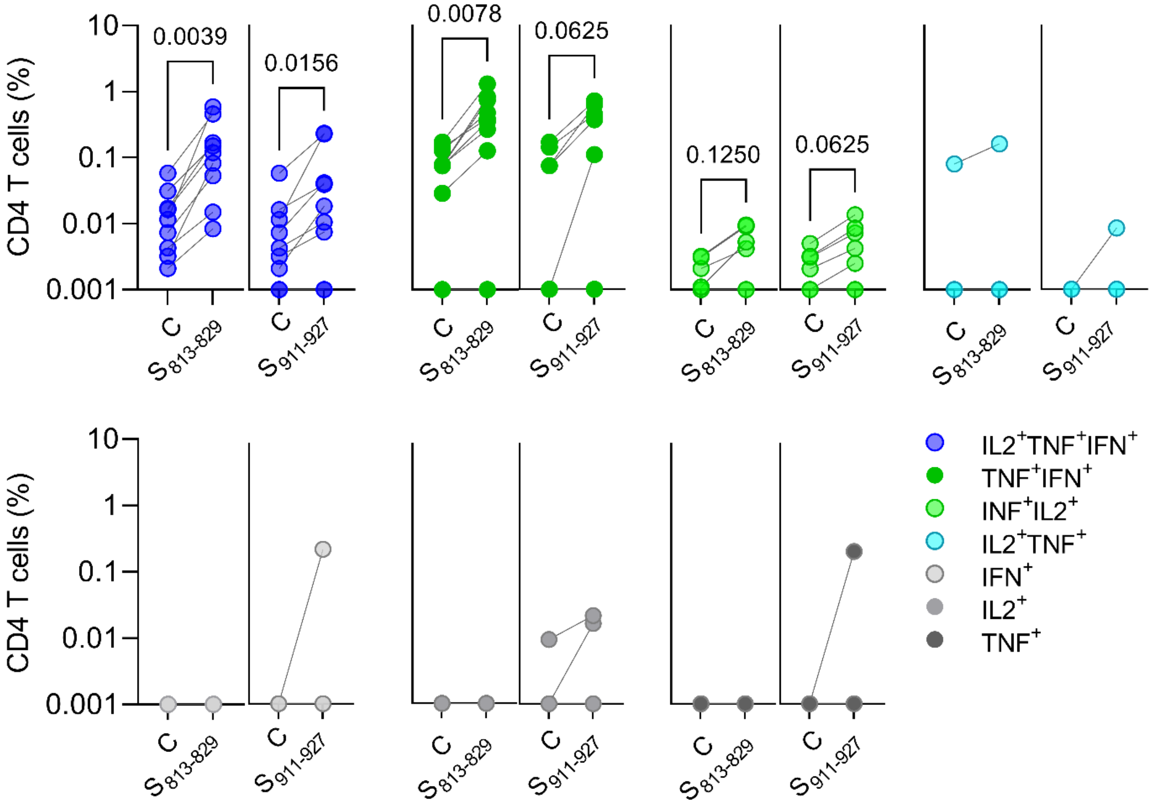

Supplementary Figure 3. Analysis of SARS-CoV-2-specific T cell responses in individuals three years after initial COVID-19 vaccination. CD4 T cell reactivity to SARS-CoV-2 $S_{813\text{-}829}$ and OC43 $S_{911\text{-}927}$. PBMCs isolated from $S_{813\text{-}829}$ responders (n=9) were propagated with SARS-CoV-2 $S_{813\text{-}829}$ for 10 days and assessed for cytokine production (interferon gamma (IFN-γ), interleukin (IL)-2, and tumor necrosis factor alpha (TNF-α) by intracellular cytokine staining after restimulation with SARS-CoV-2 $S_{813\text{-}829}$, CoV OC43 $S_{911\text{-}927}$ or no antigen as control (C). Boolean analysis was applied to discriminate seven different subtypes with indicated cytokine combinations. Frequencies below the cut-off for positivity were set to 10^{-3}. Wilcoxon matched-pairs signed rank test was used to compare CD4 T cell responses upon peptide stimulation versus negative control.

Supplementary Table 1. Characteristics of study participants

Cohort	Subcohort	Number donors	Age median (IQR)	Age range	Gender (f/m)	Severe	Mild	Wks between s.o./vac and sample collection weeks (IQR)*
Total cohort								
	Patients	72	55 (33-64)	21-80	27/45	52	20	3.6 (3.0-4.7)
	Unexposed†	18	n.a.	18-70	4/14	n.a.	n.a.	n.a.
	Vaccinated	44	41 (31-52)	20-60	35/9	n.a.	n.a.	2.4 (2.3-2.9)
HLA-matched cohort								
	Patients	55	54 (29-66)	21-80	19/36	37	18	4.0 (3.0-4.7)
	Unexposed†	16	n.a.	18-70	4/12	n.a.	n.a.	n.a.
	Vaccinated	31	47 (34-53)	19-59	23/8	n.a.	n.a.	2.4 (2.3-3.0)
Follow-up 6-12 months§								
	Convalescents	13	26 (22-29)	21-32	5/8	1	12	24.7 (24.3-25.4)
	Vaccinated	21	51 (41-54)	26-58	18/3	n.a.	n.a.	13.6 (12.9-15.3)
Follow-up 3 years#								
	Vaccinated/ Convalescents	21	52 (44-56)	23-61	16/5	0	21	35 (29-59)

*Weeks between symptom onset (s.o.) of last SARS-CoV-2 infection or last vaccination and blood sample collection. †SARS-CoV-2 unexposed, samples collected in 2013-2019. §Blood samples were obtained at 6 months after SARS-CoV-2 infection and 12 months after the second vaccine dose (all vaccinated individuals received booster vaccination 13.6 weeks prior to sample collection). #Three-year follow-up blood samples were obtained at a median of 3.5 years (IQR, 3.4-3.5 years) after the second vaccine dose. n.a., not available.

Supplementary Table 2. Frequencies of best predicted HLA-DR and -DP alleles for $S_{813-829}$ or $S_{1002-1018}$ in the study cohort

HLA II allele	Frequency %	Percentile rank	
		$S_{813-829}$	$S_{1002-1018}$
DPA1*01:03/DPB1*04:01	59%	0.57	3.60
DPA1*01:03/DPB1*02:01	26%	1.30	4.60
DPA1*01:03/DPB1*04:02	25%	1.00	1.60
DPA1*02:01/DPB1*04:01	10%	0.63	5.30
DRB1*15:01	22%	46.00	0.81

Predicted binding scores for HLA II alleles are expressed as percentile rank. A value <2 is considered a high binding score.

Supplementary Table 3. Paired analysis of cytokine responses in short-term PBMC lines

	Propagated with SARS-CoV-2 $S_{813-829}$			Propagated with OC43 $S_{911-927}$		
Cytokine	C*	$S_{813-829}$*	p-value	C*	$S_{911-927}$*	p-value§
INF-γ	0.24	0.83	0.0062	0.21	0.47	0.0015
IL-2	0.09	0.22	0.0200	0.09	0.19	0.0224
TNF-α	0.47	1.00	0.0053	0.39	0.68	0.0032

*Cytokine expression (average % of CD4 T cells) in short-term PBMC lines propagated with SARS-CoV-2 $S_{813-829}$ or OC43 $S_{911-927}$ after restimulation with the homologous peptide or unstimulated control. §Paired t-test was used to compare cytokine responses between restimulated and unstimulated controls. C, unstimulated control. INF-γ: interferon gamma, IL-2: interleukin 2, TNF-α: tumor necrosis factor alpha

Supplementary Table 4. SARS-CoV-2 specific non-conserved peptides

Aa position	Aa sequence	Aa position	Aa sequence
162-178	SANNCTFEYVSQPFLMD	120-136	VNNATNVVIKVCEFQFC
253-269	DSSSGWTAGAAAYYVGY	239-255	QTLLALHRSYLTPGDSS
414-430	QTGKIADYNYKLPDDFT	337-353	PFGEVFNATRFASVYAW
442-458	DSKVGGNYNYLYRLFRK	344-360	ATRFASVYAWNRKRISN
449-465	YNYLYRLFRKSNLKPFE	351-367	YAWNRKRISNCVADYSV
526-542	GPKKSTNLVKNKCVNFN	386-402	KLNDLCFTNVYADSFVI
631-647	PTWRVYSTGSNVFQTRA	708-724	SNNSIAIPTNFTISVTT
666-682	IGAGICASYQTQTNSPR	750-766	SNLLLQYGSFCTQLNRA
932-948	GKIQDSLSSTASALGKL	799-815	GFNFSQILPDPSKPSKR
974-990	SSVLNDILSRLDKVEAE	827-843	TLADAGFIKQYGDCLGD
1079-1095	PAICHDGKAHFPREGVF	897-913	PFAMQMAYRFNGIGVTQ

Peptides used in this study were 17 amino acids (aa) in length.

Supplementary Table 5. Univariate analysis of factors associated with infection outcome

Variable	Coefficient (ß)	95% Confidence interval (CI)	R^2	p-value (two-tailed)
Viral load*				
Age (years)	0.028	0.006 to 0.050	0.138	0.013
Gender	0.155	-0.796 to 1.106	0.003	0.744
Days since symptom onset	-0.005	-0.064 to 0.054	0.001	0.868
$S_{813-829}$-specific T cells§	-1.039	-1.583 to -0.495	0.261	<0.001
SI-specific T cells§	-0.414	-1.463 to 0.635	0.015	0.430
SII-specific T cells§	-1.291	-2.389 to -0.193	0.121	0.022
S-specific T cells§	-1.049	-2.221 to 0.123	0.074	0.078

*log vRNA copies; §log SFU/10^6 PBMCs

Supplementary Table 6. Univariate analysis of factors associated with hospitalization

Variable	Odds ratio	95% Confidence interval (CI)	Nagelkerke R^2	p-value (two-tailed)
Hospitalization				
Age (years)	1.201	1.091 - 1.322	0.783	<0.001
Gender	1.083	0.330 - 3.561	0.0	0.895
Days since symptom onset	1.006	0.939 - 1.078	0.001	0.865
$S_{813-829}$-specific T cells*	0.425	0.179 - 1.007	0.107	0.052
SI-specific T cells*	8.648	1.696 - 44.086	0.205	0.009
SII-specific T cells*	1.796	0.415 - 7.772	0.016	0.434
S-specific T cells*	4.164	0.794 - 21.834	0.080	0.092
Viral load§	4.904	1.411 - 17.042	0.376	0.012

* log SFU/10^6PBMCs; §log vRNA copies

Supplementary Table 7: Individual vaccination and infection histories

Sample ID	Age (years)	Sex f/m	Vaccine doses						SARS-CoV-2 omicron infections*		
			V1	V2	V3	V4	V5	V6	Inf #1	Inf #2	Inf #3
B0001	52	f	P	P	M	P	BIVBA.5	XBB.1.5	/	/	/
B0005	33	m	P	P	P	XBB.1.5	/	/	/	/	/
B0006	58	m	P	P	P	P	XBB.1.5	/	BA.5	/	/
B0007	49	f	P	P	P	XBB.1.5	/	/	BA.5	/	/
B0012	50	f	P	P	P	BIVBA.5	XBB.1.5	/	/	/	/
B0013	41	f	P	P	M	P	/	/	/	/	/
B0016	54	f	P	P	M	P	/	/	XBB	/	/
B0017	59	f	P	P	P	/	/	/	XBB	/	/
B0020	35	f	P	P	P	/	/	/	/	/	/
B0021	31	f	P	P	P	P	/	/	/	/	/
B0027	48	m	P	P	P	P	BIVBA.5	XBB.1.5	BA2.86	/	/
B0031	55	f	P	P	P	P	/	/	BA.5	XBB	/
B0032	57	f	P	P	P	XBB.1.5	/	/	BA.5	/	/
B0034	59	m	P	P	P	/	/	/	BA.5	/	/
B0036	48	f	P	P	P	/	/	/	BA.5	BA.5	XBB
B0042	61	f	P	P	P	P	BIVBA.5	XBB.1.5	XBB-JN.1§	/	/
B0043	44	m	P	P	P	BIVBA.5	/	/	XDK.4.1	/	/
B0045	56	m	P	P	P	/	/	/	BA.5	/	/
B0048	57	f	P	P	P	BIVBA.5	/	/	BA.2	XBB	/
B0049	24	f	P	P	P	BIVBA.5	/	/	XBB	/	/
B0051	56	f	P	P	P	P	XBB.1.5	/	BA.1	/	/

Abbreviations: m, male; f, female. V1-V6, Vaccine doses 1-6. *Infecting SARS-CoV-2 variant confirmed by VirSNiP assay and/or whole genome sequencing. §The variant circulating at the time of RT-PCR diagnosis is indicated. P: Monovalent Pfizer-BioNtech Comirnaty® BNT162b2 (WT); BIVBA.5: Pfizer-BioNtech Bivalent (WT/BA.5) BNT162b2 BA.5; M: Monovalent Moderna Spikevax® mRNA-1273 (WT). XBB.1.5: Monovalent Novavax Nuvaxovid® XBB.1.5. WT, wild-type.
